# Single-dose pharmacokinetics, tolerability, and physiologically based pharmacokinetic modeling of Fazamorexant in Chinese patients with hepatic impairment and in healthy controls

**DOI:** 10.3389/fphar.2026.1814155

**Published:** 2026-04-24

**Authors:** Qingmei Li, Min Wu, Cuiyun Li, Xingxing Huang, Lu Jin, Wenjuan Deng, Ruwei Wang, Jiajia Mai, Hong Zhang

**Affiliations:** 1 Department of Pediatric Nephrology, Children’s Medical Center, The First Hospital of Jilin University, Jilin, China; 2 Phase I Clinical Research Center, The First Hospital of Jilin University, Jilin, China; 3 Yangtze River Pharmaceutical Group Co., Ltd., Taizhou, China; 4 National Key Laboratory of Advanced Drug Formulations for Overcoming Delivery Barriers, Taizhou, China

**Keywords:** Fazamorexant, hepatic impairment, insomnia, pharmacokinetics, safety, YZJ-1139

## Abstract

**Objectives:**

YZJ-1139 (Fazamorexant) is a dual orexin receptor antagonist in development for adult insomnia. This study assessed the impact of hepatic impairment on Fazamorexant’s pharmacokinetic safety, and tolerability to guide clinical dosing.

**Methods:**

This study used a non-randomized, open-label, single-dose design. Patients with mild or moderate hepatic impairment (Child-Pugh class A or B) and healthy subjects (n = 8 per group) received a single oral 20 mg dose of Fazamorexant.

**Results:**

In total, 24 participants were enrolled and completed the study. In subjects with mild hepatic impairment, the geometric mean ratios (90% CI) of Fazamorexant plasma C_max_, AUC_0–t_, and ${\text{AUC}}_{0–\infty }$ relative to those with normal hepatic function were 97.82% (77.41%–123.60%), 145.22% (96.83%–217.79%), and 145.43% (97.70%–216.47%), respectively. In moderate impairment, the corresponding values for C_max_, AUC_0–t_, and ${\text{AUC}}_{0–\infty }$ were 104.68% (82.85%–132.28%), 153.41% (102.29%–230.07%), and 154.50% (103.79%–229.97%). Hepatic impairment did not significantly alter the peak time. Fazamorexant was generally well tolerated.

**Conclusion:**

Fazamorexant showed good safety and minimal impact of hepatic impairment on C_max_, but AUC increased by approximately 50% in patients with mild or moderate hepatic impairment.

## 1 Introduction

Insomnia is the most common clinical sleep disorder, characterized by persistent difficulty falling asleep, staying asleep, and poor sleep satisfaction (Sutton, 2021; Perlis et al., 2022; Shaha, 2023). A large-sample cross-sectional study involving 51,774 adults conducted in Guangdong Province, China, from October to December 2022 revealed a weighted prevalence of insomnia of 24.8% among the study population. Insomnia was significantly associated with both depression and anxiety (Cao et al., 2017; Dopheide, 2020; Xu et al., 2022; Zhong et al., 2022; Shan et al., 2024). Current treatments, such as benzodiazepines and melatonin receptor agonists, have limited efficacy and safety (Schroeck et al., 2016; Krone et al., 2023; Porosnicu Rodriguez et al., 2023; Matheson et al., 2024). Orexin receptor antagonists represent a novel class of insomnia drugs that promote sleep by suppressing the wakefulness drive mediated by the orexin system (Wu et al., 2022; Muehlan et al., 2023; Rocha et al., 2023). YZJ-1139 (Fazamorexant), a dual orexin receptor antagonist, is being developed for adult insomnia, particularly sleep onset and maintenance difficulties.

Fazamorexant is rapidly absorbed orally. Within the dose range of 2–80 mg, the time to peak concentration (T_max_) is 1 h (0.625–1.25 h). The elimination half-life (t_1/2_) is approximately 1.90–3.13 h; it exhibits rapid systemic clearance and is extensively metabolized primarily by CYP3A4, with no accumulation observed after repeated dosing (Ni et al., 2025). No major or pharmacologically active metabolites were detected. The unbound fraction (fu) is 0.004, and plasma protein binding is 99.6%. Fazamorexant exhibits clinically significant drug–drug interactions with itraconazole and rifampin: coadministration with itraconazole increases its AUC by > 7-fold, whereas coadministration with rifampin decreases its AUC by >90%. As a mild CYP3A4 inhibitor, it increases midazolam AUC by ∼30% (data not published). Fazamorexant represents a promising new treatment option for patients with insomnia.

This study aimed to evaluate the safety and pharmacokinetic (PK) profile of Fazamorexant in patients with mild or moderate hepatic impairment compared with healthy controls, in accordance with regulatory guidance (Haertter et al., 2025). The recommended clinical dosage is 20 mg (maximum 40 mg). The highest tolerated single dose of Fazamorexant in healthy subjects was 120 mg (6 times the recommended clinical dose and 3 times the maximum recommended dose). Given the unknown metabolic profile of fazamorexant in patients with hepatic impairment, long-term multiple-dose administration poses unacceptable safety risks. Fazamorexant exposure increases approximately linearly with dose and shows no accumulation; therefore, a single-dose study is appropriate to evaluate the impact of hepatic impairment. Accordingly, a single 20-mg dose was administered in the clinical study. The results provide guidance for the safe and rational use of Fazamorexant in patients with hepatic impairment.

## 2 Subjects and methods

### 2.1 Study population

Key inclusion criteria: Adults aged 18–70 years, BMI 18–32 kg/m^2^, and creatinine clearance ≥60 mL/min. Hepatic impairment group: Patients with chronic liver disease or cirrhosis due to primary causes (e.g., hepatitis B, hepatitis C, autoimmune or alcoholic liver disease), Child-Pugh Grade A or B, on a stable treatment regimen for at least 14 days before dosing, without need for dose adjustment or ongoing therapy. Healthy controls: Normal hepatic function confirmed by medical history, physical examination, laboratory tests, ultrasound, and ECG. Mean age and body weight within ±10 years and ±10 kg of the hepatic impairment group; gender distribution was consistent across groups, with a maximum difference of one participant per gender.

Key exclusion criteria: QTcF >470 msec in males or >480 msec in females during screening; use of CYP3A4 enzyme inducers or inhibitors within 1 month (or five half-lives) prior to dosing. Ineligible participants with hepatic impairment: alanine aminotransferase or aspartate aminotransferase >10 × ULN; absolute neutrophil count <0.75 × 10^9^/L; hemoglobin <60 g/L; alpha-fetoprotein >100 ng/mL.

This was an open-label, parallel-group clinical study conducted at a single center in China from March to April 2024 in accordance with the principles of Good Clinical Practice.

### 2.2 Study design

This Phase I, single-dose, open-label, parallel-group study evaluated the effect of hepatic impairment on the pharmacokinetics and tolerability of Fazamorexant (Clinical Trial Registration Number: NCT06671509). Eight participants with mild hepatic impairment, eight with moderate impairment, and eight matched healthy controls were enrolled.

All participants were hospitalized and fasted overnight for at least 10 h before receiving a single 20 mg oral dose of Fazamorexant as a tablet on Day 1. The batch number is 24022731. Water intake was prohibited within 1 h before and after dosing. Safety was monitored *via* vital signs and 12-lead ECG. Following safety assessment, participants were discharged on Day 3, with a telephone follow-up conducted on Day 7.

### 2.3 Pharmacokinetic sampling and analysis

Blood samples (3 mL) were collected in tubes containing the anticoagulant sodium heparin for pharmacokinetic analysis at time points of 0 (60 min before administration), 0.25 h, 0.5 h, 0.75 h, 1.0 h, 1.5 h, 2.0 h, 3.0 h, 4.0 h, 6.0 h, 8.0 h, 12.0 h, 24.0 h, 36.0 h, and 48.0 h. At both the 1-h and the 12-h post-administration time points, an additional venous blood sample (1 mL) was collected to determine the free drug concentration of Fazamorexant. Samples were centrifuged for 10 min at 2 °C–8 °C and 1,500 × g to separate plasma from blood cells and other components.

All samples were stored at −80 °C until analysis was performed using a validated analytical method based on liquid chromatography–tandem mass spectrometry (LC-MS/MS), with human plasma as the matrix for calibration standards and quality control samples. The linearity range for Fazamorexant in plasma was 8–9,000 ng/mL.

Pharmacokinetic parameters included primary parameters such as maximum plasma concentration (C_max_), area under the curve from time zero to the last measurable concentration (AUC_0–t_), and area under the curve from time zero to infinity ( ${\text{AUC}}_{0–\infty }$ ); and secondary parameters including T_max_, t_1/2_, apparent clearance (CL/F), and apparent volume of distribution (Vz/F), all calculated using WinNonlin version 8.3 (Certara, Princeton, NJ, USA) *via* noncompartmental analysis based on actual sample collection times.

### 2.4 Development of the physiologically based pharmacokinetic (PBPK) model

A PBPK model for Fazamorexant was developed and rigorously validated using the open-source software PK-Sim® (version 12.0, part of the Open Systems Pharmacology Suite) (Sun et al., 2024). Comprehensive methodological details are provided in the Supplementary Materials, and the final model parameters are summarized in Table S1. Statistical analyses were performed using R (R Foundation for Statistical Computing, Vienna, Austria) and RStudio (version 2023.09.1–494, Posit, Inc., Boston, USA).

### 2.5 Safety assessments

The safety population included participants who received the study drug. Safety assessments were based on vital signs, 12-lead ECGs, laboratory tests (hematology, clinical chemistry, coagulation, urinalysis), and physical examinations. Treatment-Emergent Adverse Events (TEAEs) were classified by severity using CTCAE v5.0 and assessed for relationship to the study drug.

### 2.6 Statistical analysis

Descriptive statistics summarized demographic, safety, and pharmacokinetic (PK) data. Continuous variables are presented as mean ± SD; categorical variables as frequency and percentage. Primary PK parameters of Fazamorexant ( ${\text{AUC}}_{0–\infty }$ , AUC_0–t_, C_max_) were log-transformed and analyzed using ANOVA with hepatic function as a fixed effect. Results are expressed as geometric least-squares means with 90% confidence intervals (CIs) across hepatic function groups. T_max_ differences between groups were assessed using nonparametric tests. Analyses were performed in SAS version 9.4 (SAS Institute Inc.).

## 3 Results

### 3.1 Participants

A total of 41 subjects were screened for this trial, and 24 subjects were enrolled to receive Fazamorexant treatment—8 in the normal hepatic function group (healthy controls), eight in the mild hepatic impairment group, and eight in the moderate hepatic impairment group. All 24 subjects completed the trial. The demographic characteristics and baseline variables of the participants are summarized in Table 1.

**TABLE 1:** Demographic data of the study subjects.

Baseline parameter	Mild hepatic impairment (N = 8)	Moderate hepatic impairment (N = 8)	Normal hepatic function (N = 8)
Age (years)	57.3 ± 6.18	55.5 ± 5.83	53.0 ± 3.93
Body weight (kg)	70.14 ± 8.622	73.66 ± 9.217)	68.25 ± 4.743)
BMI (kg/m^2^)	26.4 ± 2.39	25.9 ± 3.27	25.0 ± 1.51
Race, n (%)	​	​	​
Han	8 (100)	7 (87.5)	8 (100)
Other	0	1 (12.5)	0
Sex, n (%)	​	​	​
Male	5 (62.5)	8 (100)	6 (75.0)
Female	3 (37.5)	0	2 (25.0)

Data are expressed as mean ± standard deviation or numbers of subjects (%).

### 3.2 Pharmacokinetics of fazamorexant

**FIGURE 1:**
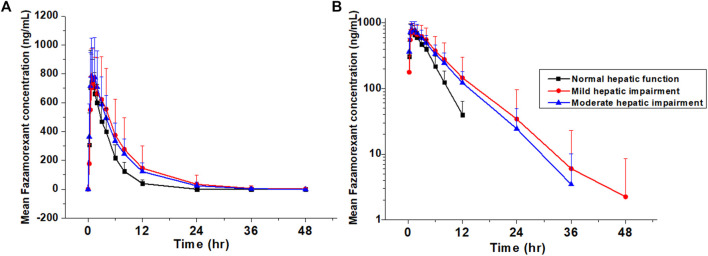
Mean plasma Fazamorexant concentrations after administration in participants with normal hepatic function and in participants with mild or moderate hepatic impairment **(A)** linear scale **(B)** semi-log scale.

All collected data from participants were included in the pharmacokinetic and statistical analyses. The mean plasma concentration–time profiles and pharmacokinetic parameters of Fazamorexant in individuals with normal, mild, or moderate hepatic impairment are shown in Figure 1 and Tables 2, 3. Following a single 20-mg dose of Fazamorexant, the drug was rapidly absorbed, with C_max_ achieved at 0.876–1.25 h and t_1/2_ ranging from 2.348 to 4.441 h. In subjects with mild hepatic impairment, the geometric mean ratios (90% CIs) of Fazamorexant plasma C_max_, AUC_0–t_, and ${\text{AUC}}_{0–\infty }$ relative to those with normal hepatic function were 97.82% (77.41%–123.60%), 145.22% (96.83%–217.79%), and 145.43% (97.70%–216.47%), respectively; CL/F was 68.76% (46.20%–102.36%). In moderate hepatic impairment, the corresponding values were 104.68% (82.85%–132.28%), 153.41% (102.29%–230.07%), and 154.50% (103.79%–229.97%) for exposure parameters, and 64.73% (43.48%–96.35%) for CL/F. Compared with normal hepatic function, Fazamorexant C_max_ was similar in mild and moderate hepatic impairment, whereas ${\text{AUC}}_{0–\infty }$ increased by 45.43% and 54.50%, and CL/F decreased by 31.24% and 35.27%, respectively. No significant difference in T_max_ was observed across groups (P = 0.418).

**TABLE 2:** Pharmacokinetic characteristics of Fazamorexant.

PK parameters	Mild hepatic impairment (N = 8)	Moderate hepatic impairment (N = 8)	Normal hepatic function (N = 8)
C_max_ (ng/mL)	792.250 ± 220.321	857.375 ± 271.342	800.500 ± 163.652
T_max_ (h)	1.250 (0.75, 3.01)	1.000 (0.50, 1.50)	0.876 (0.50, 2.00)
AUC_0-t_ (h*ng/mL)	5,985.326 ± 4,496.613	5,508.496 ± 2,161.050	3,449.747 ± 912.610
${\text{AUC}}_{0-\infty }$ (h*ng/mL)	6,172.207 ± 4,535.507	5,710.988 ± 2096.889	3,592.422 ± 1,008.607
t_1/2_ (h)	3.925 ± 1.735	4.441 ± 1.512	2.348 ± 0.316
CL/F (mL/h)	4,640.470 ± 2,457.350	3,969.781 ± 1,546.451	5,937.653 ± 1,568.484
V_z_/F (mL)	21,457.065 ± 5,537.633	23,300.912 ± 6,049.125	19,582.100 ± 3,263.340
Fup (%)^#^	0.356 ± 0.211	0.363 ± 0.240	0.344 ± 0.293

Data are expressed as mean ± standard deviation or median (minimum, maximum); # Fup: fraction of unbound protein.

**TABLE 3:** Ratios for pharmacokinetic parameters of Fazamorexant in subjects with hepatic impairment *versus* normal hepatic function.

PK parameters	Groups	Ratio 90% CI (%)
C_max_	Mild	97.82 (77.41–123.60)
​	Moderate	104.68 (82.85–132.28)
AUC_0-t_	Mild	145.22 (96.83–217.79)
​	Moderate	153.41 (102.29–230.07)
${\text{AUC}}_{0-\infty }$	Mild	145.43 (97.70–216.47)
​	Moderate	154.50 (103.79–229.97)
CL/F	Mild	68.76 (46.20–102.36)
​	Moderate	64.73 (43.48–96.35)

Linear regression analysis showed no significant association between pharmacokinetic parameters (C_max_, AUC_0–t_, and ${\text{AUC}}_{0–\infty }$ ) and hepatic function markers (albumin, total bilirubin, and prothrombin time) (P > 0.05; data not shown). The fraction of unbound protein (Fup, %) was similar across all groups (0.344–0.363).

### 3.3 PBPK modeling

Finally, we employed the PBPK model, which accurately fitted the PK curves following single-dose administration in healthy controls and participants with mild to moderate hepatic impairment from the current study. This demonstrated good predictive accuracy, with geometric mean fold errors (GMFEs) of 1.05 for AUC_0-t_ and 1.13 for C_max_ (Supplementary Figure S1; Supplementary Table S2).

### 3.4 Tolerability of fazamorexant

A total of 24 subjects were included in the safety dataset; 23 (95.8%) experienced 33 TEAEs. No serious adverse events, serious adverse reactions, or TEAEs leading to withdrawal occurred. All eight subjects (100%) in the mild hepatic impairment group experienced 11 TEAEs, nine of which were classified as adverse reactions. In the moderate hepatic impairment group, all 13 TEAEs reported by seven subjects (87.5%) were adverse reactions. All eight subjects (100%) in the normal hepatic function group experienced 9 TEAEs, all of which were adverse reactions.

Somnolence (91.7%) and drowsiness (4.2%) were the most common adverse reactions, with a combined incidence of 95.8%, consistent with Fazamorexant’s mechanism of action. Incidence rates were similar across groups. Excluding drowsiness and somnolence, other adverse reactions occurred in 12.5%, 37.5%, and 12.5% of subjects in the mild hepatic impairment, moderate hepatic impairment, and healthy control groups, respectively—slightly higher in the moderate group (Table 4). Only one subject (B002, moderate group) had a grade 3 decrease in white blood cell count (baseline grade 2; possibly related); all other TEAEs were grade 1–2. All TEAEs resolved spontaneously without treatment. Fazamorexant tablets were well tolerated in subjects with mild to moderate hepatic impairment and in those with normal hepatic function.

**TABLE 4:** Adverse events summary after administration of Fazamorexant.

Adverse events	Mild hepatic impairment (N = 8), n (%)	Moderate hepatic impairment (N = 8), n (%)	Normal hepatic function (N = 8), n (%)
Somnolence	7 (87.5)	7 (87.5)	8 (100)
Drowsiness	1 (12.5)	0	0
Decreased white blood cell count	0	2 (25)	0
Decreased neutrophil count	0	1 (12.5)	0
Sinus bradycardia	0	1 (12.5)	0
Decreased serum albumin	1 (12.5)	1 (12.5)	0
Increased serum bilirubin	0	1 (12.5)	0
Swelling of the right hand	0	0	1 (12.5)

* N = number of subjects analyzed, n = number of subjects.

## 4 Discussion

In this Phase I, single-dose, open-label, parallel-group study, the pharmacokinetics and tolerability of Fazamorexant were evaluated in participants with mild or moderate hepatic impairment, given that hepatic elimination is the primary route of Fazamorexant clearance.

Overall, a single 20-mg dose of Fazamorexant was generally well tolerated by all participants. However, no new TEAEs were reported in this study compared with previous Phase I–III studies. Most reported TEAEs were mild or moderate in intensity and resolved without intervention. The most common TEAE was somnolence (91.7%), which is consistent with the mechanism of action of Fazamorexant; no TEAEs specific to hepatic impairment were observed compared with healthy controls. The occurrence of drowsiness also supports the pharmacodynamic activity of Fazamorexant. Currently, three orexin receptor antagonists—suvorexant, lemborexant, and daridorexant—are approved worldwide for the treatment of insomnia (Khazaie et al., 2022; Riemann et al., 2023; Xue et al., 2023). Common adverse reactions include headache, somnolence, and fatigue (Xue et al., 2023). Fazamorexant shows a similar safety profile.

Fazamorexant exposure, such as AUC, was 50% higher in participants with hepatic impairment than in healthy controls, but C_max_ was similar across groups. Compared with healthy controls, Fazamorexant metabolism was slower in participants with hepatic impairment after dosing. In healthy controls, the drug concentration declined to 5% of its peak level 12 h post-dose, compared to 24 h in those with hepatic impairment. This study included 10 participants with hepatic impairment who had been receiving concomitant medications for HBV infection for at least 4 weeks before screening. Since none of these medications are CYP3A4 enzyme inhibitors or inducers, they are unlikely to affect the pharmacokinetics of Fazamorexant (Sun et al., 2024).

Hepatic impairment may alter drug pharmacokinetics by affecting metabolic enzyme activity, plasma protein binding, and hepatic blood flow. In this study, the fraction unbound was similar across all groups, indicating that plasma protein binding was not affected. The extent of PK changes depends on both the drug’s characteristics and the severity of hepatic impairment (Piscitelli et al., 2022). Rapid attainment of peak concentrations and comparable Cmax values suggest that hepatic impairment does not affect absorption. However, AUC increased by approximately 50% in participants with mild or moderate hepatic impairment, suggesting slightly reduced metabolism, although metabolic capacity was similar between these two groups. PBPK modeling showed clearances of 1.48, 1.02, and 1.01 mL/min/kg for healthy controls and those with mild and moderate hepatic impairment, respectively, supporting these findings. The PBPK model was an exploratory analysis conducted after the completion of this study to further clarify pharmacokinetic characteristics (such as clearance); it was not planned during the design stage of this study. The results of this PBPK model will provide a basis for the application of PBPK models in future similar studies.

The pharmacokinetic effects of hepatic impairment on similar drugs are as follows: for daridorexant, C_max_ remains unchanged and AUC increases by 1.6-fold in subjects with moderate hepatic impairment, and both remain unchanged in subjects with mild hepatic impairment; for lemborexant, C_max_ increases by approximately 1.5-fold and AUC by 1.25-fold in subjects with mild hepatic impairment, and C_max_ increases by 1.25-fold and AUC by 1.5-fold in subjects with moderate hepatic impairment. Fazamorexant shows a hepatic impairment profile similar to that of daridorexant (Preskorn, 2023). The final dosing regimen for participants with hepatic impairment and insomnia should be determined based on the safety and efficacy of treatment in patients with mild or moderate hepatic impairment, in conjunction with the drug exposure levels observed in this study. Thus, this study provides a foundation for developing dosing recommendations for participants with hepatic impairment and insomnia, and supports refinement of the drug label.

This study has several limitations. First, it excluded subjects with severe hepatic impairment (Child–Pugh C). Second, the small sample size may limit generalizability. Patients with hepatic impairment often have underlying diseases and concomitantly use other drugs. During the study design phase, concomitant drug use was restricted. However, complex drug interactions in real-world settings may further affect pharmacokinetics. Real-world data from patients with insomnia and hepatic impairment should be continuously collected to inform dose adjustment. Further studies with larger samples are needed to confirm the findings.

## 5 Conclusion

Similar C_max_ values and approximately a 50% increase in AUC of Fazamorexant were observed in participants with mild to moderate hepatic impairment compared with healthy controls. A single 20-mg dose of Fazamorexant was well tolerated in these participants.

## Data Availability

The original contributions presented in the study are included in the article/Supplementary Material, further inquiries can be directed to the corresponding authors.
