# Supplementary material for: Single-dose pharmacokinetics, tolerability, and physiologically based pharmacokinetic modeling of Fazamorexant in Chinese patients with hepatic impairment and in healthy controls
Source: Front Pharmacol. 2026 Apr 24;17:1814155. doi: 10.3389/fphar.2026.1814155 (PMC13153453; doi:10.3389/fphar.2026.1814155)
Supplement: Supplementary file 1 [file Supplementaryfile1.docx]

## Supplement materials

## Development and verification of the PBPK model

First, a preliminary PBPK model was constructed using the drug’s physicochemical properties and preclinical pharmacological data. Next, the Fazamorexant PBPK model was validated using pharmacokinetic data from single-dose administration of the Fazamorexant tablet formulation. The model was then verified against PK parameters obtained from studies in subjects with mild or moderate hepatic impairment. Ratios of predicted-to-observed values of Cmax and AUC_0-t_ were used to assess the model’s fit against clinical trial results. Sensitivity analyses and parameter optimization were performed to refine model accuracy. Model performance was quantitatively assessed using the GMFE for AUC_0-t_ and Cmax values (Equations 1–2). A GMFE within a twofold range was predefined as indicating successful model prediction.

$GMFE={10}^{\left( \sum\left| \log_{10} ratio \right| \right)/n}$ (Equation 1)

$ratio={{PARM}_{pred}}/{{PARM}_{obs}}$ (Equation 2)

with n=number of studies, PARM_pred=_predicted AUC_0-t_ and C_max_ value, PARM_obs_=corresponding observed AUC_0-t_ and C_max_ value

## Result

Table S1. Final PBPK model parameters for Fazamorexant.

| Parameter | Value^a^ |
| --- | --- |
| Physiochemical properties | |
| Lipophilicity | 2.8 |
| Molecular Weight (g/mol) | 432.49 |
| Halogens | F (1) |
| Fraction Unbound | 0.004 |
| Protein Binding | Albumin |
| Solubility (mg/l) | 0.17 ^b^ |
| pKa | neutral |
| Specific intestinal perm (cm/second) | 2.75E-05 ^c^ |
| Partition coefficients | Rodgers and Rowland |
| Cellular permeabilities | PK-Sim Standard |
| Specific organ perm | PK-Sim Calculated |
| Formulation | Weibull |
| Dissolution time (min) | 25 ^b^ |
| Dissolution shape | 0.66 ^b^ |
| Metabolism and Transport | |
| CYP3A4 Vmax (nmol/pmol/min) | 0.0158 |
| CYP3A4 Km (μmol/L) | 4.49 ^b^ |
| GFR | 1^c^ |

a Fazamorexant unannotated parameters are unpublished preclinical data provided by Yangtze River Pharmaceutical Group Co., Ltd., China; b Optimized value; c Assumed value.

Table S2. Observed and simulated pharmacokinetic parameters of Fazamorexant in participants with normal hepatic function and those with hepatic impairment.

| Subject | Dose (mg) | AUC_0-t_ (ng/mL*h) | | | C_max_ (ng/mL) | | |
| --- | --- | --- | --- | --- | --- | --- | --- |
|  |  | Predicted | Observed | Ratio | Predicted | Observed | Ratio |
| Normal hepatic function | 20 | 3510 | 3450 | 1.02 | 703 | 801 | 0.88 |
| Mild hepatic impairment | 20 | 5318 | 5985 | 0.89 | 722 | 792 | 0.91 |
| Moderate hepatic impairment | 20 | 5513 | 5508 | 1.00 | 747 | 857 | 0.87 |


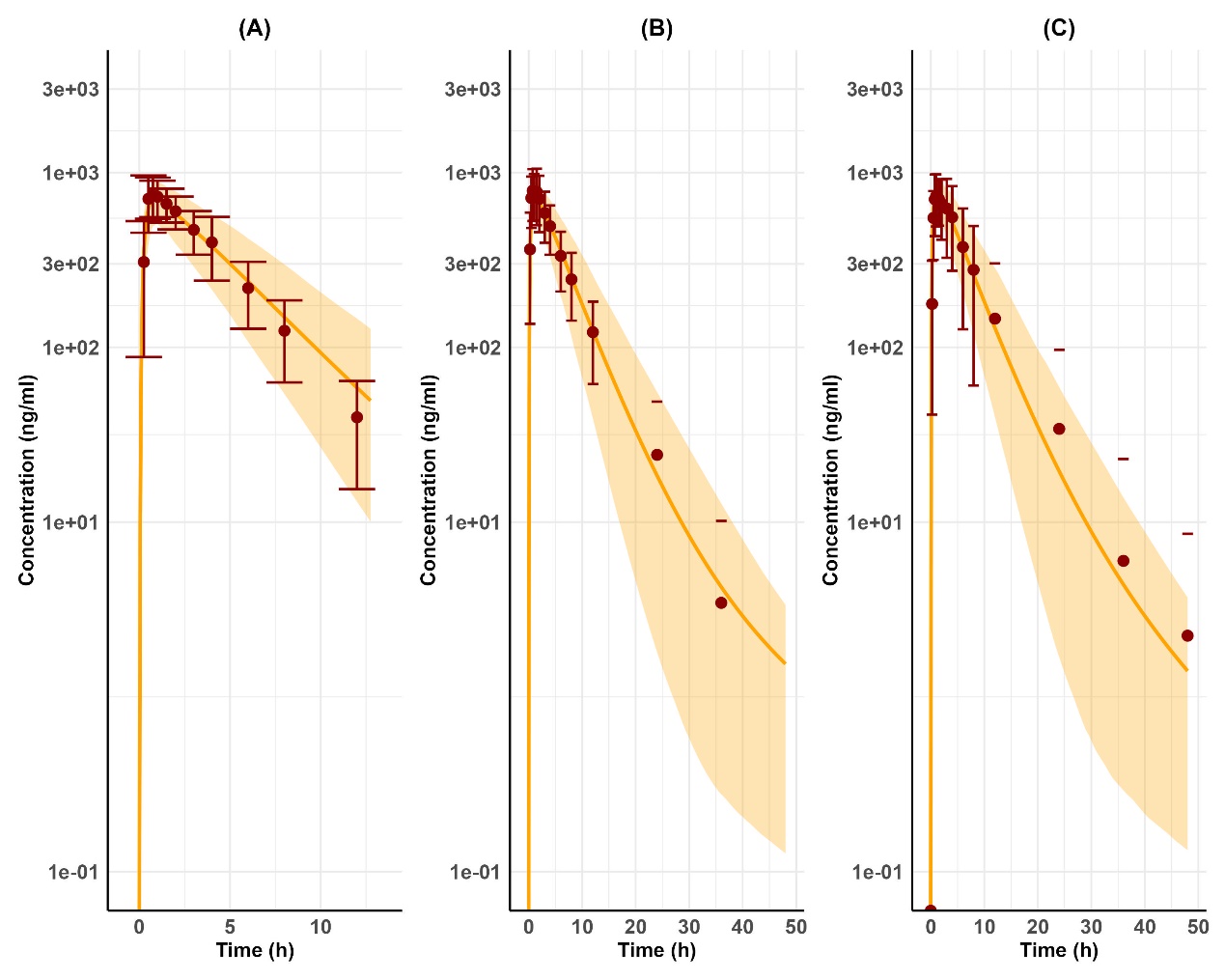


Figure S1. Simulation results of the Fazamorexant PBPK model in participants with normal hepatic function and in participants with hepatic impairment.

Solid lines represent simulated mean plasma concentrations; shaded areas represent the 5th–95th percentile range.

• Panel A: Fazamorexant 20 mg single dose in participants with normal hepatic function.

• Panel B: Fazamorexant 20 mg single dose in participants with mild hepatic impairment.

• Panel C: Fazamorexant 20 mg single dose in participants with moderate hepatic impairment.
